# Genetic Substrate Reduction Therapy: A Promising Approach for Lysosomal Storage Disorders

**DOI:** 10.3390/diseases4040033

**Published:** 2016-11-09

**Authors:** Maria Francisca Coutinho, Juliana Inês Santos, Liliana Matos, Sandra Alves

**Affiliations:** Research and Development Unit, Department of Human Genetics, INSA, National Health Institute Doutor Ricardo Jorge, Rua Alexandre Herculano, 321 4000-055 Porto, Portugal; juliana.santos@insa.min-saude.pt (J.I.S.); liliana.matos@insa.min-saude.pt (L.M.); sandra.alves@insa.min-saude.pt (S.A.)

**Keywords:** substrate reduction therapy (SRT), Gaucher disease (GD), mucopolysaccharidosis type III (MPS III; Sanfilippo syndrome), combination therapy

## Abstract

Lysosomal storage diseases are a group of rare genetic disorders characterized by the accumulation of storage molecules in late endosomes/lysosomes. Most of them result from mutations in genes encoding for the catabolic enzymes that ensure intralysosomal digestion. Conventional therapeutic options include enzyme replacement therapy, an approach targeting the functional loss of the enzyme by injection of a recombinant one. Even though this is successful for some diseases, it is mostly effective for peripheral manifestations and has no impact on neuropathology. The development of alternative therapeutic approaches is, therefore, mandatory, and striking innovations including the clinical development of pharmacological chaperones and gene therapy are currently under evaluation. Most of them, however, have the same underlying rationale: an attempt to provide or enhance the activity of the missing enzyme to re-establish substrate metabolism to a level that is consistent with a lack of progression and/or return to health. Here, we will focus on the one approach which has a different underlying principle: substrate reduction therapy (SRT), whose uniqueness relies on the fact that it acts upstream of the enzymatic defect, decreasing storage by downregulating its biosynthetic pathway. Special attention will be given to the most recent advances in the field, introducing the concept of genetic SRT (gSRT), which is based on the use of RNA-degrading technologies (RNA interference and single stranded antisense oligonucleotides) to promote efficient substrate reduction by decreasing its synthesis rate.

## 1. Introduction

The concept of substrate reduction therapy (SRT) was first suggested 20 years ago by Norman Radin [1]. Unlike enzyme replacement therapy (ERT), which was originally proposed as a general mechanism to overcome any of the lysosomal enzyme deficiencies underlying lysosomal storage disorders (LSDs), SRT was first brought to light as a potential adjunct therapy for Gaucher disease (GD) patients and nothing else. Still, soon after its principle was first enunciated, other research teams devoted efforts to the search of additional chemical inhibitors for the biosynthesis of lysosomal substrates. The ground was thus seeded for the establishment of an alternative line of work on the LSDs field, whose major focus was to decrease the biosynthesis rate of any of the accumulating substrates in order to achieve a therapeutic effect. This approach was called SRT and presently, two decades after its rationale was first established, two SRT drugs have already been approved (miglustat, Zavesca^®^ for GD and Niemann-Pick type C1 and eliglustat tartrate, Cerdelga^®^ for GD alone) and a third one is undergoing clinical trials (genistein for Mucopolysaccharidoses, MPSs; ORPHA79213 [2,3,4]). It should be noticed, though, that clinical evaluation of those same drugs has unveiled a few side effects, the most well-known being those observed for miglustat, which included osmotic diarrhea and weight loss. To overcome this issue, second generation compounds are currently under evaluation for their potential to reduce storage of glycopshingolipids (GSLs). Also under consideration is a fully molecular approach, which aims to selectively downregulate specific genes involved in the biosynthesis of accumulating substrates by using gene suppression technologies. This approach has recently been referred to as genetic SRT (gSRT; [5]).

The development of miglustat (*N*-butyl-deoxynojirimycin; *N*B-DNJ) and eliglustat tartrate as therapeutic drugs for Gaucher disease type 1 (GD 1), as well as their biochemical and molecular impact has been extensively discussed in dozens of research papers and an equivalent series of thematic reviews (see for example Venier and Igdoura [6] for a review on miglustat and Shayman [7] for a detailed overview on the development of eliglustat tartrate). Also, the use of genistein for mucopolysaccharidoses (MPSs) has been extensively discussed and reviewed over the last few years [8]. Nevertheless, a systematic review on the literature highlighting the recent developments in gSRT is still missing, and, given the promising results achieved by independent teams in a series of disorders using different RNA-degrading technologies, we believe it is not only relevant but also necessary to gather those same results and discuss them altogether. Here, we will review the use of gene suppression technologies as tools to achieve substrate reduction and will summarize and compare the results from different teams.

Gene suppression technologies for therapeutic purposes have been one of the major interests of the pharmaceutical industry in the last decade. One of the most promising seems to be RNA interference (RNAi), whose efficacy has already been evaluated at a cellular level, effectively downregulating a large number of target genes whose products are involved in disease. RNAi applications are emerging, particularly in the fields of oncology, viral infections, diabetes, and neurological, cardiovascular, bone-related, and ocular diseases [9], with several clinical trials ongoing or in the recruitment phase [10]. In general, RNAi clinical trials are progressing well. With several trials in Phase III development, RNAi therapeutics are likely to be approved by the U.S. Food and Drug Administration (U.S. FDA) within the next few years. The approval of the first RNAi therapeutic should pave the way for approval of other targets [11]. Also, other antisense oligonucleotides (AOs) for gene knockdown have successfully been tested for different disorders and are now under clinical evaluation (Table 1). Nevertheless, reports on the use of RNA-based technologies as therapeutic tools for LSDs are still scarce. RNAi was tried to inhibit glucosylceramide synthase, one of the key enzymes involved in the synthesis for GD [12]. Other interesting results were obtained when small interfering RNA (siRNAs) were used to reduce glycosaminoglycan (GAGs) synthesis in MPS IIIA mice [13] and in MPS IIIC patient fibroblasts [14]. To the best of our knowledge, only one single stranded AO (ssAO) approach has been attempted for substrate reduction so far: the suppression of muscle glycogen synthase 1 synthesis for Pompe disease [15].

Here we will summarize the striking results of RNA-degrading technologies as tools to achieve substrate reduction for LSDs, highlighting their therapeutic potential, as well as the challenges they raise, namely the need to develop more stable and potent gene repression systems with lower levels of added therapeutic, which are necessary before such approaches reach the clinic.

## 2. RNA Interference (RNAi) as a Mechanism to Promote Substrate Reduction

**Figure 1:**
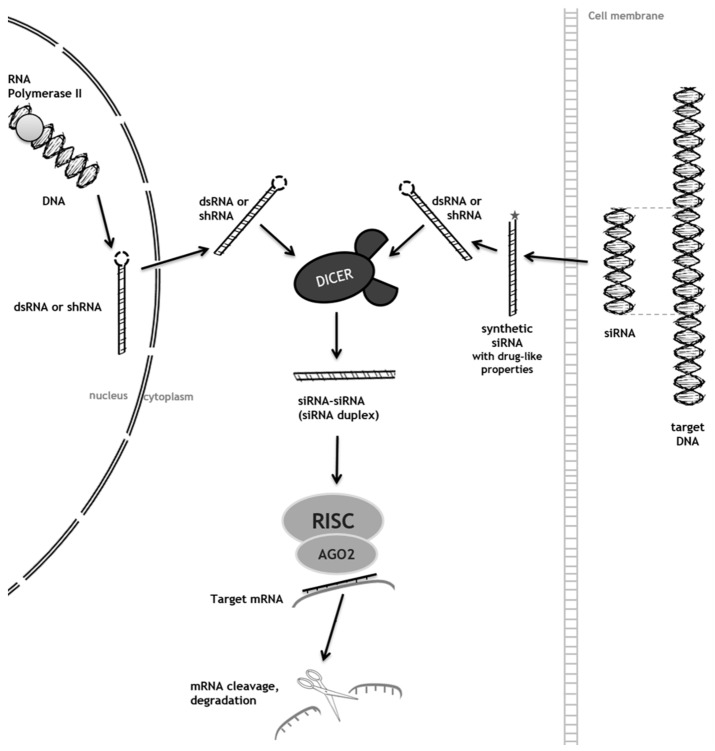
Natural process and therapeutic mechanism of RNAi (RNA interference).

RNAi is a rather new technology that has revolutionized the way that researchers study mammalian gene expression. It has had significant impact on the ease, speed, and specificity with which the loss of gene function analysis can be done in mammalian cells and animal models. Thus, it is becoming the method of choice for loss of function studies. Being based on a naturally occurring post-transcriptional gene silencing process, RNAi technology takes advantage of the cell’s natural machinery, facilitated by siRNAs, to effectively knockdown the target gene’s expression (Figure 1; [16]).

Reports on the use of RNAi to achieve substrate(s) reduction in LSDs by artificially decreasing their biosynthesis rate are limited. Originally, it was used to inhibit the genes implicated in glycosphingolipid metabolism [17], being subsequently tested for its ability as a gSRT for GD [12], MPS IIIA [13,18] and, more recently, MPS IIIC [14].

### 2.1. RNAi-Mediated SRT for Gaucher Disease

Proof-of-principle on the potential of RNA-degrading technologies as effective tools to achieve significant reduction of the levels of accumulated substrate(s) in LSDs cell lines was provided in 2006 when Diaz-Font and co-workers [12] assessed the potential of an artificially-induced RNAi mechanism to reduce storage in GD.

Even though GD is an uncommon metabolic disorder, it is the most prevalent LSD, with an estimated birth frequency of 1/50,000 in the Caucasian population [19]. It is an autosomal recessive disorder caused by mutations in *GBA*—the gene encoding for β-Glucocerebrosidase (GCase), with more than 400 mutations described (www.hgmd.org; HGMD database Professional 2016.3 Release).

GD is classically divided into three variants according to the absence or presence and progressivity of neuropathic disease. All variants have differing degrees of enlargement of liver and spleen, anemia, thrombocytopenia, and skeletal disease. These can range from mild to very severe within each type, although the rate of progression generally is greater in younger patients. Also, the degrees of visceral organ involvement are not concordant in patients. For example, massive involvement of the liver and spleen is not necessarily accompanied by severe bone disease. The reverse is also true. In addition, this classification is not age dependent, but depends on the primary involvement of the central nervous system (CNS) by GD in any age. GD 1 patients are free of primary CNS involvement. The variability of the phenotype of visceral manifestations ranges from severe fatal disease in the first two decades to essentially asymptomatic nonagenarians. Gaucher disease types 2 and 3 have primary CNS neuronopathic involvement. Types 2 and 3 represent a continuum of disease phenotypes that differ primarily in their rates of CNS and visceral disease progression. This continuum encompasses phenotypes leading to death *in utero*, or in the first few days of life, to rapidly progressive CNS and visceral diseases that are fatal in the first years, to more slowly progressive (yet severe) CNS (with mild to severe visceral disease) deterioration over a period of 2–3 years to decades [20,21,22,23,24].

Currently, there are two main disease-specific therapies available for Gaucher disease: ERT (with imiglucerase, Cerezyme^®^; velaglucerase alfa, VPRIV^®^, or taliglucerase alfa, Elelyso^®^) and SRT (with miglustat Zavesca^®^ or eliglustat tartrate, Cerdelga^®^). ERT has proved successful for the treatment of most type 1 GD patients even though it has some limitations and disadvantages, such as the high cost and the lifelong dependence. Also, the lack of effect on neurological symptoms hinders its use by patients suffering from either type 2 or 3 GD. In turn, the use of miglustat has been associated with some side effects. Therefore, additional therapeutics are under investigation for this disease. One of them is RNAi mediated by siRNAs, which was used to inhibit GCase gene expression.

After transcription, double-stranded RNAs (dsRNAs) or short-hairpin RNAs (shRNAs) exit the nucleus and reach the cytoplasm (natural process; *left side of Figure 1*). Alternatively, when generated for therapeutic purposes, small-interfering RNAs (siRNAs) are designed to correspond to a target gene as they are chemically synthesized with the following drug-like properties: stability and conjugation for delivery (grey star, *right side of Figure 1*). Regardless of the mechanism by which they reach the cytoplasm, once they get there, shRNAs are processed by DICER (double-stranded RNA (dsRNA) endoribonuclease), an endonuclease, into mature siRNA duplexes. One of the double-stranded siRNA is loaded into a RISC (RNA-induced silencing complex), which contains the AGO2, a protein of the Argonaute (AGO) protein family. Then, the siRNA guide strand recognizes and links to the target site by base pairing, causing mRNA cleavage and degradation (adapted from [16]).

This approach was first tested in 2006 by a group in Spain. Diaz-Font and coworkers [12] designed four different siRNAs for the human *GCS* gene and subsequently transfected them into HeLa cells. After doing so, the authors observed a clear reduction of *GCS* RNA levels. They also performed enzyme *GCS* activity assays to confirm inhibition at the protein level and the results were positive. Altogether, the results seemed promising and completely in line with the hypothesis that RNAi would be an effective method for genetic inhibition of *GCS*. The most interesting results, however, came from the evaluation of a reduction in glucosylceramide synthesis. Similar results were obtained when plasmids expressing shRNAs (targeting the same sequences) were transfected into the cells. The inhibition of the mouse homolog *Ugcg* gene was also achieved using a siRNA that targeted both human and mouse sequences [12].

Also noteworthy, a partial inhibition of the *GCS* gene could also be used for other pathologies with glycosphingolipid accumulation in the brain, such as Tay–Sachs, GM1-gangliosidosis, or even Niemann–Pick type C or some of the MPSs, for which no current treatment is available [12].

Even though promising, the use of siRNAs as therapeutic tools requires several important issues related with its usage to be addressed, namely off-target effects, efficacy, delivery, and immune system activation. However, we understand that future research will overcome all these difficulties allowing for their generalized used in the short term.

### 2.2. RNAi-Mediated SRT for Mucopolysaccharidoses

Amongst LSDs, the Mucopolysaccharidoses (MPSs) are a major subgroup, with crucial historical relevance, which are caused by impaired degradation of GAGs; they were formerly known as mucopolysaccharides. Depending on the individual enzyme deficiency, one or more pathways may be affected. Currently, the MPS group comprises 11 different conditions, caused by individual deficiencies of enzymes that catalyze GAGs stepwise degradation.

Generally, it is possible to say that MPSs are chronic and progressive diseases characterized by a huge clinical and biochemical heterogeneity: a single enzymatic deficiency may underlie severe, intermediate or mild forms of the disease; also, different biochemical deficiencies may generate similar clinical phenotypes. Still, it is possible to say that MPSs do share several clinical features, even though in different degrees of severity. The most severe forms of the disease present in the newborn period or even *in utero*. Overall, there is a predominance of somatic manifestations including skeletal and joint deformities (dysostosis multiplex), which result in a characteristic abnormal facies and chronic, constant, and acute pain. Typical symptoms also include organomegaly, with liver and/or spleen being the most affected organs. As the disease progresses, patients suffer from a relentless decline of the clinical, physical, and mental picture, eventually losing mobility and developing feeding and breathing difficulties. Finally, some MPSs are also characterized by severe neurological degeneration (MPS III, for example). Whatever the case, disease severity seems to depend on several factors, including the type of substrate accumulated, the accumulation levels, and the tissues and/or cells in which that accumulation occurs. At a molecular level, it is possible to say that the type of causal mutation harbored by each patient may frequently influence the severity of the disease in that particular individual, as demonstrated by our team amongst others [25,26,27].

As with most LSDs, there is no fully effective treatment for MPSs. In fact, besides hematopoietic stem cell transplantation (HSCT), the only MPSs with specific therapies so far are types I (ORPHA579), II (ORPHA580), IVA (ORPHA582), and VI (ORPHA583), where the condition is fought by perfusion of a recombinant enzyme. This approach, however, fails to correct the neurological symptoms, since recombinant enzymes are unable to cross the blood brain barrier (BBB) [28]. Consequently, the most severe forms of MPS I and II (where there is strong neurological involvement) are not efficiently treated through ERT. Therefore, MPSs are amongst those LSDs that need further investment and special attention by the different research teams in order to face the major challenge of creating a therapy that targets not only the somatic manifestations, but also the neurological ones. Naturally, a possible approach would be not only to correct the enzymatic defect but also to reduce the levels of biosynthesis of the accumulating substrate by SRT. Chemical SRT has already been attempted for MPS diseases through the use of genistein (for a detailed revision on the subject see [29]). Still, this treatment has not been approved so far, and it has a series of disadvantages and secondary effects; further clinical studies are needed before it can be approved by the regulators. The other possibility, which we will address here, was to achieve substrate reduction by gSRT.

As most biochemical pathways that give rise to GAGs are already known and the majority of involved genes have been cloned, it seemed reasonable to take advantage of the naturally-occurring RNAi mechanism and model it in such a way that it targets the enzymes responsible for GAGs biosynthesis. This approach was attempted by several teams that targeted different genes on the biosynthetic pathway that produce the different GAGs that accumulate in MPS diseases. Once again, their rationale was simple: if they decreased GAGs synthesis by RNAi of specific genes, they would actively reduce substrate accumulation and slow down the pathology.

Thus, early in this decade, studies on a very interesting genetic model for substrate reduction therapy indicated efficient substrate reduction and improved efficacy of ERT when RNAi was used as a mechanism to inhibit GAGs biosynthesis. MPS type IIIA fibroblasts were transfected with four siRNAs for *XYLT1*, *XYLT2*, *GALTI,* and *GALTII* genes. By real-time PCR, the authors observed a significant decrease in the mRNA levels of all target genes. Furthermore, the respective protein levels were also reduced in cells treated with the designed siRNAs, from 30% to 87% relative to the negative control. To investigate the effects of gene silencing on GAG synthesis, Dziedzic and colleagues [13] measured the incorporation of ^35^S sulphate in siRNA-transfected cells and observed a considerable impairment in GAGs synthesis. Later, a study performed by the same team showed that inhibition of GAGs synthesis in cells treated with two siRNAs together was more effective than the use of single siRNAs [30].

By the same time, Kaidonis et al. [18] used RNAi technology to specifically target two genes involved in Heparan sulphate (HS) synthesis: *EXTL2* and *EXTL3*. HS is the GAG accumulated in MPS III (Sanfilippo syndrome) patients. In the synthetic pathway of HS, *EXTL* genes play a crucial role since *EXTL2* and *EXTL3* are key proteins involved in HS chain elongation. They have shown that shRNAs directed to *EXTL2* significantly reduced endogenous target gene expression in a reporter gene assay. This approach has also significantly decreased HS synthesis and reduced lysosomal storage of this GAG in an MPS IIIA cell line. One of the three shEXTL3s decreased endogenous *EXTL3* expression and subsequent HS synthesis. In addition, the incorporation of the shRNAs into a stable lentiviral expression system also resulted in reduction of target gene expression in a reporter gene assay [18].

Later, Chmielarz and co-workers [31] compared the effects of different treatment methods on the regulation of GAGs storage in various cell lines of MPS. They studied the effect of ERT alone (laronidase; Aldurazyme^®^), the effect of different siRNAs alone, and the effect of both treatments combined. As they expected, ERT was more effective than siRNA treatment in reducing GAGs storage in all studied cell lines. When a combination of these two treatments was employed, different results were obtained depending on the cell line. The combination of these two therapeutic approaches can be less or more pronounced than that obtained with either method alone. It is important to notice that neither laronidase nor siRNAs cause cytotoxic effects in most MPS cell lines. However, some of those effects may occur in particular cell lines [31].

More recently, Canals and co-workers evaluated the efficiency of four siRNAs to inhibit *EXTL2* and *EXTL3* gene expression. They used fibroblasts of two different patients, which were observed from day 3 to day 14 after transfection, compared to cells treated with negative control siRNAs using *SDHA* and *HPRT* as endogenous genes. Around 90% of mRNA inhibition capacity was observed for the siRNAs used. GAGs synthesis was quantified through the evaluation of the cell incorporation of a ^35^S sodium sulfate medium, as previously described by Dziedzic, when evaluating the potential of *XYLT1*, *XYLT2*, *GALTI,* and *GALTII* gene inhibition as a tool for Sanfilippo gSRT [13]. Fibroblasts showed a decrease of about 30% to 60% in the incorporation of ^35^S sulfate after 3 days of transfection when compared to control samples. Using an imunocytochemistry assay, they observed that, after a 3-day treatment with a siRNA for *EXTL2*, patients’ fibroblasts showed a significant reduction in HS accumulation [14].

Altogether, these results highlight the feasibility of a gSRT approach for MPS diseases, clearly demonstrating that positive effects on the levels of GAG accumulation may be achieved whatever the step of the biosynthetic pathway one might target. This is particularly important since it paves the way for the potential use of a single compound to treat a series of pathologies.

## 3. Antisense Oligonucleotides (AOs) as Tools to Achieve Substrate Reduction

Besides double-stranded siRNA and shRNA oligonucleotides, other AOs with a single-stranded chain can be used for gene knockdown expression.

Antisense technology was demonstrated for the first time by Paterson and colleagues in 1977 [32], who used recombinant single-stranded DNA molecules to inhibit mRNA translation. Since then, remarkable progress has been made in oligonucleotide drug development; currently, single-stranded AOs (ssAOs) that alter gene expression for therapeutic benefit are used through a variety of mechanisms, including inhibition of 5’ cap formation, steric blocking of protein translation, RNA splicing modulation, and activation of RNase H to degrade the target mRNA [33,34]. A few ssAO drugs have been approved already by the U.S. FDA for use in the clinic: Formivirsen (Vitravene^®^), Pegaptanib (Macugen^®^), and Mipomersen (Kynamro^®^). Formivirsen is an antiviral ssAO used for repression of cytomegalovirus mRNA translation. It gained U.S. FDA approval for intraocular treatment for cytomegalovirus retinitis in immunosuppressed patients [33,35,36]. Pegaptanib is a 28-base ssAO developed to specifically bind to and block the activity of vascular endothelial growth factor (VEGF)_165_, the VEGF isoform primarily responsible for abnormal vascular growth and permeability in wet age-related macular degeneration (AMD), while sparing the physiological isoform VEGF_121_ [33,37]. Finally, mipomersen is a systemically-delivered ssAO which targets the apolipoprotein B-100 mRNA, a critical component of atherogenic lipid particles, and indicated for the treatment of heterozygous familial hypercholesterolemia [33,36,38]. Furthermore, several other ssAO drugs have reached advanced clinical trials (Table 1).

**Figure 2:**
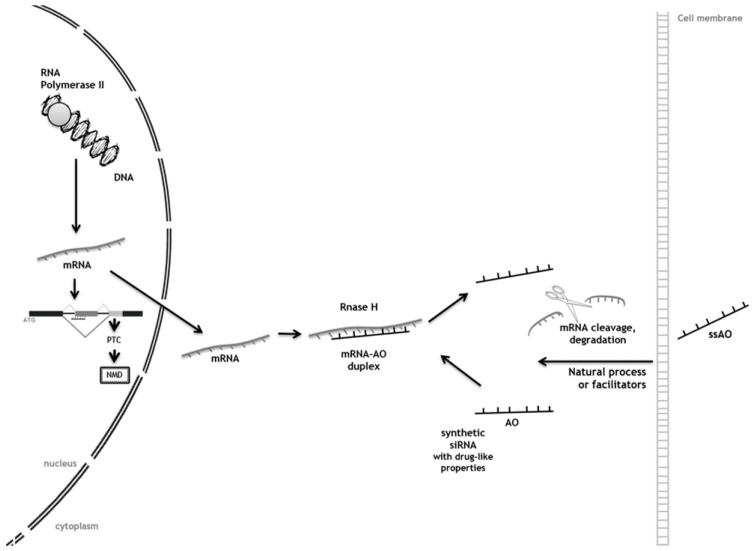
Single stranded antisense oligonucleotide (AO) pathways for gene knockdown: two examples.

AOs targeted to an early out-of-frame exon induce exon skipping, thus causing a frameshift. The resulting premature termination codon (PTC) triggers nonsense mediated mRNA decay through a process referred to as forced splice-dependent nonsense mediated decay (FSD-NMD; *left side of Figure 2*). Other AOs interact with the cell membrane and use different mechanisms, natural processes, or facilitators to enter the cell. In the cytoplasm, AOs can bind the target RNA through the AO-mRNA duplex formation, which activates RNase H enzyme and then cuts the mRNA preventing the synthesis of the protein (*right side of Figure 2*).

### Antisense Oligonucleotide-Mediated SRT for Pompe Disease

Recently, an AO-mediated gene suppression approach was evaluated for its potential as a substrate reduction therapy for Pompe disease (Glycogen storage disease type II; OMIM#232300), a LSD caused by a deficiency of acid alpha-glucosidase (GAA; EC 3.2.1.20). Typically, such deficiency results in a progressive intralysosomal accumulation of glycogen, which is more evident in cardiac and skeletal muscles, even though the enzyme is deficient in all tissues. In classic cases of Pompe disease, affected children are prostrate and markedly hypotonic with large hearts. The tongue may also be enlarged. The liver is rarely enlarged, except as a result of heart failure, and hypoglycemia and acidosis do not occur. Death usually occurs in the first year of life in the classic form of the disorder and cardiac involvement is striking. Indeed, the Dutch physician who originally described this disorder, Joannes Cassianus Pompe, reported it as ‘idiopathic hypertrophy of the heart’ [39], and ‘cardiomegalia glycogenica’ may also be used as a synonym. Even though ERT by injection of a recombinant human GAA (rhGAA) is already available for Pompe, there are still some aspects of the disease that elude treatment. In fact, albeit the infusion of rhGAA prolongs survival, improves cardiomyopathy and walking ability while stabilizing pulmonary function [40,41,42,43,44,45,46], residual muscle weakness and hearing loss are still evident after ERT. Also, the risk of developing arrhythmias, dysphagia, and osteopenia remains undiminished [47].

Taking this into account, Nicholas Clayton and co-workers [15] designed an alternative treatment option for patients with Pompe disease, which held potential to ameliorate other symptoms once combined with the available ERT. In order to promote substrate reduction, they designed a phosphorodiamidate morpholino oligonucleotide (PMO)-based method to invoke exon skipping and premature stop codon (PTC) usage in the gene which codes for muscle glycogen synthase 1 (*Gys1*). Their assumption that such a procedure would result in a decrease of the accumulation of lysosomal glycogen was grounded on previous studies by Douillard-Guilloux et al. [48]. These authors had already addressed the modulation of glycogen synthesis by RNAi towards a novel SRT approach and showed that by knocking out *Gys1* in a Pompe disease mouse model a profound decrease in lysosomal glycogen accumulation was achieved together with a reduction in lysosomal size in primary myoblasts. In the same study, it was shown that *Gys1/GAA* double knockouts had better tetanic force generation and fatigue resistance compared to *GAA* knockout animals. The concept of *Gys1* protein knockdown was further examined through the use of siRNA designed to degrade *Gys1* mRNA after viral delivery using AAV2/1, resulting in a strong inhibition of *GYS* expression and a significant decrease in cytoplasmic and vacuolar glycogen accumulation [48]. Additionally, it has been demonstrated that treatment with rapamycin suppresses *GYS* activity in Pompe mice leading to lower glycogen levels in skeletal muscle. When used in combination with rhGAA infusions, it lowered glycogen levels in the skeletal muscle and diaphragm more effectively than either agent alone [49].

The approach designed by Clayton [15] was based on the systemic administration of the designed PMO conjugated to a cell penetrating peptide (GS-PPMO; gelatin-silica cell penetrating peptide conjugated to phosphorodiamidate morpholino oligonucleotide) in order to facilitate delivery in Pompe mice. Candidate PMOs were first tested by direct injection into the tibialis anterior muscle of mice followed by electroporation. Two weeks later, *Gys1* mRNA levels were quantified. Twelve PMOs were tested and two resulted in what appeared to be a substantial reduction in *Gys1* mRNA. One PMO sequence in particular targeted the skipping of exon 6 and was evaluated further after conjugation. It resulted in an efficient, dose-dependent decrease of glycogen synthase transcripts in both the quadriceps and the diaphragm. Nevertheless, no statistically significant differences were seen in the liver. In the heart, mRNA response was seen only at the higher dose tested. Still, it is important to notice that, concomitant to the observed decreases in target mRNA, the protein activity and levels were diminished and, most importantly, those reductions resulted in significant decreases in the aberrant accumulation of lysosomal glycogen. Also worth mentioning, treatment had no overt toxicity, thus supporting the idea that SRT by GS-PPMO-mediated inhibition of muscle specific glycogen synthase represents a viable therapeutic strategy for Pompe disease, which certainly deserves further development.

To the best of our knowledge, this is the only report on the use of AO technology as a tool to achieve substrate reduction in an LSD. Still, as the authors themselves have already pointed out, the development of PPMO for human use is significantly challenged by the renal toxicity, which has been observed in primate studies for a similar drug candidate developed for Duchenne muscular dystrophy [50]. This toxicity was likely due to the cationic nature of the peptide and was observed at doses required for efficient exon skipping in the primate musculature. Those previous studies were conducted with the same prototypic peptide that was used in the *GYS1* studies here reviewed. Thus, safer peptide conjugates will need to be developed. Another possibility will be to refine the dosing regimen before these results can be translated into a clinical therapy. Nevertheless, these results demonstrate proof of concept for *GYS1* knockdown as a therapeutic approach for Pompe disease, clearly supporting the need for additional research on the subject and further highlighting the potential of gene suppression technologies as straightforward methods to achieve substrate reduction in this group of pathologies.

## 4. Conclusions

We have reviewed a series of studies providing proof of principle on the use of RNA-degrading technologies (RNAi and AOs) as efficient tools to achieve gSRT in LSDs models. Some of those studies were performed in model cell lines overexpressing the targets [12] and others in patients’ cell lines [13,14,18,31]. Effects were evaluated at mRNA and protein levels but, most importantly, on the levels of the major accumulated substrate(s) in each target pathology, and results were encouraging, as extensively demonstrated in the previous sections. However, if siRNAs are to be used as therapeutic tools, there are a series of relevant, important problems that should be addressed. First, one would have to either continuously provide the siRNA(s) to the cells or compel the cells to produce them. This can be achieved by the transfection of DNA vectors bearing shRNAs [51]. That is why several authors have already tested the efficacy of shRNAs as gSRT triggers instead of the chemically synthesized easy-to-handle double-stranded siRNAs, showing significant decreases in substrate accumulation when used with the more technically challenging vector-based molecules. In addition to high potency and sustainable effects using low copy numbers, there are several advantages associated with the use of shRNA molecules to induce RNAi for therapeutic purposes, these include their lower potential to activate immune response and induce inflammation and toxicity, together with fewer off-target effects [52]. Still, this strategy will face the same problems already found by the more “classical” gene therapy approaches, in other words, transfecting the gene encoding the missing enzyme. Identically, the chemical structures of ssAOs also face a number of important constraints that still need to be overcome, namely those related with delivery, tissue-uptake, drug-induced toxicities, body clearance, and safety.

In general, however, the results from all the studies here reviewed were quite promising, since substrate reduction could be achieved in every single one. It is also important to mention that there are other technologies allowing for the sequence-specific inhibition of gene function, such as clustered regularly-interspaced short palindromic repeats (CRISPR) that have the potential to be used as therapeutic modalities [53]. While no studies have been published so far on the use of this RNA-guided mechanism that, as RNAi, may cause the cleavage of its respective sequence complementary targets, we believe it is only a matter of time until CRISPR is attempted as a therapeutic mechanism in the LSDs field given its ability to readily alter germlines, including those in humans. Indeed, CRISPR is already making strong inroads into crop production [53,54].

An additional advantage of gSRT approaches is the fact that they hold the potential to act at a neurological level since the RNA-degrading effectors are small molecules capable of crossing the BBB. Still, translation into clinics requires proper vectors for in vivo deliverance and suitable animal models to test the approach before trials.

Delivery of AOs may be assessed in vivo by evaluating the efficacy of non-viral vectors able to cross the gap between cell culture and animal models and, thus, allowing stable complexation with the nucleic acid protection and deliverance. For example, chemically synthesized siRNAs are most effectively introduced into cells using electroporation or commercially available lipid reagents, but these methods are poorly suited for in vivo delivery. Nevertheless, recent developments by independent teams have demonstrated the feasibility of systemic administration of either chemically modified or complexed AOs [9,55,56], holding promise for the translation of RNA-based gene knockdown therapies to the clinic. Different teams have been assessing different complexation methods with functional [57] and/or different vectors: exosomes [58] including lipid nanocarriers such as pegylated immunoliposomes (PILs; [59]), stable-nucleic-acid-lipid-particles (SNALPs; [60]), or polyhydroxyalkanoate-based nanovehicles [61]. Also, local delivery to the CNS, a region that is difficult to deliver drugs to due to the BBB, is being addressed, with promising results. First preliminary evidence that in vivo downregulation of specific genes by RNAi could work at the CNS level came from studies in rats and mice using invasive local delivery methods [62]. Lately, however, evidence is accumulating on the successful brain delivery of si/shRNAs using specifically designed vectors and/or modifications that include the use of enzyme-sensitive lipid nanoparticles [63], carbosilane dendrimers [64], cholesterol modifications [65], and recombinant fusion proteins [66]. Also the pharmaceutical industry is investing in developing BBB-directed vectors. biOasis Technologies Inc., for example, has developed a family of vectors called Transcend that take advantage on the existence of specific receptors and transport systems, which are highly expressed at the BBB to provide essential substances to brain cells. The Transcend vectors comprise a full-length protein (Melanotransferrin) and may be used to facilitate receptor mediated drug delivery into the brain to treat CNS disorders. Recently, the application of this new peptide vector to siRNA and ongoing studies addressing the brain delivery of Iduronate 2-sulfatase (I2S) for the treatment of Hunter Syndrome in knockout mice was discussed at the Brains4Brain society meeting and its results were quite promising [67]. Nevertheless, once those molecules have been complexed, other issues have to be addressed, such as their efficacy as a whole and their potential toxicity internalization, genotoxicity and cell viability. Finally, their effect in vivo will have to be evaluated in proper animal models before we move to clinical trials. This last step is not as challenging in the LSD field as in other fields because for a large percentage of LSDs, there are a number of animal models, either engineered or naturally occurring, that nicely mimic patients’ symptoms. As LSDs are monogenic disorders, they are a unique model to study not only the endo-lysosomal system, but also, for example, neurodegeneration itself. In fact, CNS involvement is a hallmark of many of these disorders. Thus, they present a one-of-a-kind opportunity to check the effect of each single protein. In fact, although the accumulation of intermediate degradation products is due to a single gene defect, this affects the whole cellular metabolic system causing neuronal death, apoptosis, or necrosis in advanced stages of disease. In order to unveil the processes underlying the phenomena, different teams have created different animal models for LSDs, from the usual mice to sheep and cats [68]. These animals can now be used to test for the efficacy of any gSRT effector molecule.

Currently, access to therapies is hindered by cost and geography. This major challenge remains and should also be considered from the research point of view. The development of small molecular substrate synthesis inhibitors may address the geographic boundaries [28]. Furthermore, such therapeutic approaches may be used not only as a standard to ameliorate the symptoms of LSD patients who suffer from pathologies without available ERT, but also as a complement to ERT (or any other available therapy such as the use of chaperones or any improved gene therapy protocol) for those already under treatment, in an approach commonly referred to as combination therapy [5]. Hopefully, one such approach will help to diminish damage and slow down pathology.

For example, in the future, Pompe patients may be treated effectively with a combination of rhGAA and a PPMO-based glycogen synthase inhibitor complimentary to the human target. GS-PPMO showed reasonable activity at reducing *Gys1* mRNA, protein, and activity in skeletal muscle, including the diaphragm, while being less effective in the heart, while rhGAA is considerably more effective at clearing glycogen from the heart than from skeletal muscle [15]. This suggests that the merits of the two treatment approaches are complimentary and potentially useful in some combination paradigm. Still, further testing is needed to assess the durability of GS-PPMO efficacy and check whether it is possible to employ a longer interval between administrations.

Ultimately, the same principle may be extended to other LSDs. Amongst other advantages, this kind of approach would likely allow for the creation of multifunctional mixtures, as different diseases share the same accumulating substrates. Therefore, instead of a ‘one-compound-to-one-disease’ approach, gSRT (as SRT in general) may pave the way for a ‘one-compound-to-treat-several-diseases’ era, reducing therapy costs and increasing the number of patients with available therapeutic options.

## Figures and Tables

**Table 1:** Summary table of clinical trials currently active for selected small interfering RNA (siRNA) and antisense therapeutics (adapted from [10]).

Drug	Target	Condition	Phase	Status
*RNAi*				
DCR-MYC	Myc	Solid tumors Hepatocellular carcinoma	I	Recruiting
			I/II	Recruiting
ALN-TTRSC (Revusiran)	Transthyretin	TTR-mediated familial amyloidotic cardiomyopathy	I	Completed
			II	Active
			III	Active
ALN-CC5	Complement component C5	Paroxysmal nocturnal Hemoglobinuria	I/II	Active
ALN-AS1	ALAS-1	Acute intermittent porphyria	I	Recruiting
ALN-PCSSC	PCSK9	Hypercholesterolemia	I	Completed
ALN-TTR02 (Patisiran)	Transthyretin	TTR-mediated amyloidosis	II	Active
			III	Active
ALN-AT3SC	Antithrombin	Hemophilia A/B	I	Recruiting
TKM-080301	Polo-like kinase 1	Hepatocellular carcinoma Neuroendocrine tumors	I	Active
			I/II	Recruiting
TKM-100802	Ebola genome	Ebola virus	I/II	Terminated
QPI-1007	Caspase 2	Primary angle-closure glaucoma	II	Completed
RXI-109	CTGF	Hypertrophic scar keloid excision surgery	II	Completed
			II	Completed
Atu027	PKN3	Pancreatic ductal carcinoma	I/II	Completed
SYL040012	Β2-adrenergic receptor	Ocular hypertension	II	Completed
*Antisense*				
Mipomersen *	ApoB 100	Heterozygous familial hypercholesterolemia atherosclerosis	III	Completed
			III	Completed
ISIS-TTR_RX_	Transthyretin	Familial amyloid polyneuropathy	III	Active
ISIS-ApoC-III_RX_	ApoCIII	Hypertriglyceridemia	II	Completed
ISIS-DMPK_RX_	DMPK	Myotonic dystrophy type 1	I/II	Recruiting
ISIS APO(a)-L_RX_	Apoliprotein (a)	Elevated lipoprotein (a)	I	Completed
			II	Completed
Curtirsen	Clusterin	Non-small cell lung cancer Prostate cancer	III	Recruiting
			III	Active

* Already approved by the U.S. FDA under the commercial designation Kynamro^®^.
